# More than a duologue: In-depth insights into epitranscriptomics and ferroptosis

**DOI:** 10.3389/fcell.2022.982606

**Published:** 2022-09-12

**Authors:** Justin Chak Ting Cheung, Guangzheng Deng, Nathalie Wong, Yujuan Dong, Simon Siu Man Ng

**Affiliations:** ^1^ Department of Surgery, Prince of Wales Hospital, The Chinese University of Hong Kong, Shatin, Hong Kong SAR, China; ^2^ CUHK-Shenzhen Research Institute, The Chinese University of Hong Kong, Shatin, Hong Kong SAR, China

**Keywords:** ferroptosis, epitranscriptomics, iron metabolism, lipid peroxidation, reactive oxygen species

## Abstract

Beyond transcription, RNA molecules are enzymatically modified to influence the biological functions of living organisms. The term “epitranscriptomics” describes the changes in RNA strands aside from altering the innate sequences. Modifications on adenosine (A) are the most widely characterized epitranscriptomic modification, including N^6^-methyladenosine (m^6^A), N^1^-methyladenosine (m^1^A), polyadenylation, and adenosine-to-inosine (A-to-I) RNA editing, and modifications on other nucleotides seem to be fewer, such as N^7^-methylguanosine (m^7^G), 5-methylcytosine (m^5^C), and pseudouridine (Ψ). These changes on the RNA strand surface, exclusively by their RNA-modifying proteins (RMPs), are reported in various biological phenomena, including programmed cell death (PCD). One necro-biological phenomenon that has been observed for long but has started to gain heed in recent years is “*ferroptosis*.” The phospholipid peroxidation by polyunsaturated-fatty-acid-containing-phospholipid hydroperoxyl (PLOOH) radicals destroys membrane integrity due to a series of mechanisms. The Fenton reaction, constituting the final Haber–Weiss reaction that is less recognized, collaboratively leading to the conversion of polyunsaturated fatty acid (PUFA) to PLOOH, is the etymological origin of ferroptosis. However, it is with increasing evidence that ferroptotic signaling is also intervened by epitranscriptomic modifications, although the truth is still ambiguous. We attempted to delineate some up-to-date discoveries on both epitranscriptomics and ferroptosis, bringing up the fundamentals to address any potential connection between the two. Next, we discussed whether a duologal relationship, or more, exists between the two, taking the ROS level and iron status into consideration. Lastly, we surveyed future perspectives that would favor the understanding of these topics.

## Graphical Abstract

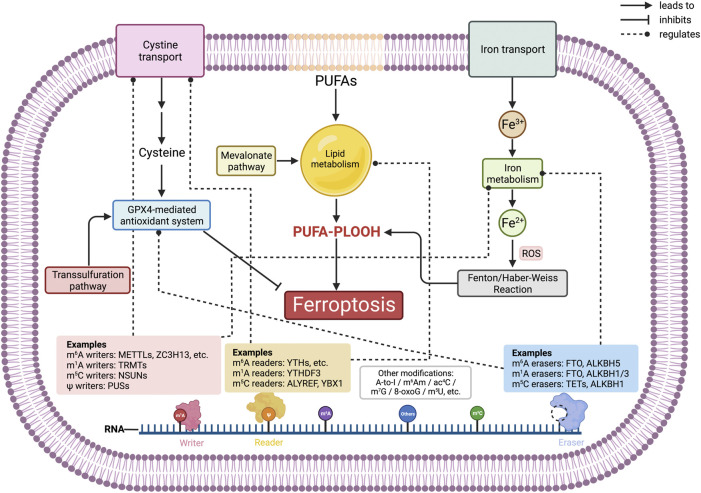

## Introduction

The RNA world theory hypothesized that every living matter originated from RNA as the entity of evolutionary heredity, in lieu of DNA (Rana & Ankri, 2016). After that, a myriad of scientists have boosted our awareness of RNA through their work and established the principles underlining the *Central Dogma* of molecular biology. Nevertheless, beyond transcription, RNA molecules can also be enzymatically modified, building a new field of epitranscriptomics that is currently under intense interest. These modifications are reported in various physiological and pathological processes, which are reviewed brilliantly elsewhere, such as tRNA modifications in the role of development (Frye et al., 2018) and transcriptional and chromatin regulation by m^6^A (Wei & He, 2021) (Shi et al., 2019). Moreover, their respective RNA-modifying proteins (RMPs) are also the targets for the investigation of epitranscriptomic regulations (Shi et al., 2019). Specific to oncological research, these RNA-modifying processes are often hijacked in cancers to acquire pro-survival advantages, and aberrant epitranscriptomic modifications have been implicated in resistance to programmed cell death (PCD). Ferroptosis, a new type of PCD denoted by an iron-dependent lethal accumulation of lipid peroxides, has started to gain heed in recent years. The complexity in ferroptotic signaling has indeed offered more opportunities for potential therapeutic manipulations in treating cancer. We attempted to delineate the up-to-date discoveries on both epitranscriptomics and ferroptosis, bringing up the fundamentals to address any potential connection between the two. Next, we discussed whether a duologal relationship, or more, exists between the two, taking the ROS level and iron status into consideration. Lastly, we surveyed future perspectives that would favor the understanding of these topics.

## Beyond transcriptomics: epitranscriptomics

RNA comprises several kinds of modifications on the transcripts that constitute the epitranscriptome. The enzyme-mediated covalent modifications on RNA, also termed epitranscriptomic modifications, experienced an arduous period after the pioneering discovery of pseudouridine (ψ) in 1951 by Davis and Ellen as the first epitranscriptomic modification (Davis & Allen, 1957). After the early work from Perry & Kelley, (1974) proving the existence of an mRNA epitranscriptomic modification in mouse L-cells, it has then become clearer that the life cycle of an mRNA transcript does not merely experience transcription but also posttranscriptional processing such as 5′-capping, poly-adenylation, and most importantly in the context of this article, epitranscriptomic modifications.

Epitranscriptomic modifications are observed in both coding mRNA transcripts (Frye et al., 2018) (Gilbert et al., 2016) and non-coding RNA, such as long non-coding RNA (lncRNA) (Yin et al., 2021), microRNA (miRNA) (Konno et al., 2019), and transfer RNA (tRNA) (Pereira et al., 2018). Dysregulated epitranscriptomic modifications on both coding mRNA and tRNA have been intuitively considered signatures in pathologies (Destefanis et al., 2021) (Suzuki, 2021) (Yang et al., 2020). Specifically, posttranscriptional editing determines the RNA fate through mediating cellular processes, including alternative splicing (Xue et al., 2021), nonsense-mediated mRNA decay (Li et al., 2019a), and translation (Ranjan & Leidel, 2019). Extending to biological functions, the epitranscriptome has built its niche in physiological regulation, which is exemplified by circadian rhythm regulation by A-to-I editing catalyzing the ADAR enzyme family (Terajima et al., 2017), GBM-associated protein expression upregulated by METTL3 via SOX2 (Visvanathan et al., 2018), and poor prognostic characterization through the IGF2BP/SOX2/METTL3 axis in CRC (Li et al., 2019b).

### Epitranscriptomic signatures and RNA-modifying proteins

According to MODOMICS, an RNA modification database constructed by Boccaletto et al. few years ago, documented RNA modifications have now raised to 144 (Dunin-Horkawicz et al., 2006), and the upsurge continues due to improved sequencing techniques and other technological advancements. To date, discussions on RNA modifications mainly revolve around the well-characterized ones, including N^6^-methyladenosine (m^6^A), 5-methylcytosine in RNA (m^5^C), N^1^-methyladenosine (m^1^A), and pseudouridine (Ψ). Others like 5-hydroxymethylcytosine (5-hmC), N^4^-acetylcytidine (ac^4^C), and adenosine-to-inosine editing (A-to-I) are only registered with unknown or unspecified functions. Moreover, MODOMICS covers the related diseases and pathways (Dunin-Horkawicz et al., 2006), with sequential updates at regular intervals (Machnicka et al., 2013) (Boccaletto et al., 2018), leading to more attention directed to the rising role of RNA modifications contributing to the nuanced transcriptomic homeostasis from clinicians and scientists (Song et al., 2020).

The fate of an mRNA transcript is determined by a series of events posttranscriptionally, and one of such crucial processes is epitranscriptomic modifications. In general, the process of mRNA epitranscriptomic editing relies on three major types of RNA-modifying proteins (RMPs):

1) writers that deposit RNA modifications, for e.g., methyltransferase-like (METTL) enzyme family members, zinc finger CCCH-type containing 13 (ZC3H13), and VIRMA/KIAA1429 for m^6^A, TRMT family members for m^1^A, ADARs for A-to-I editing, and NSUNs for m^5^C;
2) erasers that remove the epitranscriptomic modifications, for e.g., fat mass- and obesity-associated protein (FTO) for m^6^A and AlkB homologs (ALKBH) for m^1^A, m^6^A, and m^5^C;
3) readers that are recruited and recognize the modifications to alter the fate of mRNA transcripts, for e.g., YT521-B homology (YTH) domain family members for m^6^A and Aly/REF export factor (ALYREF) for m^5^C.

**FIGURE 1:**
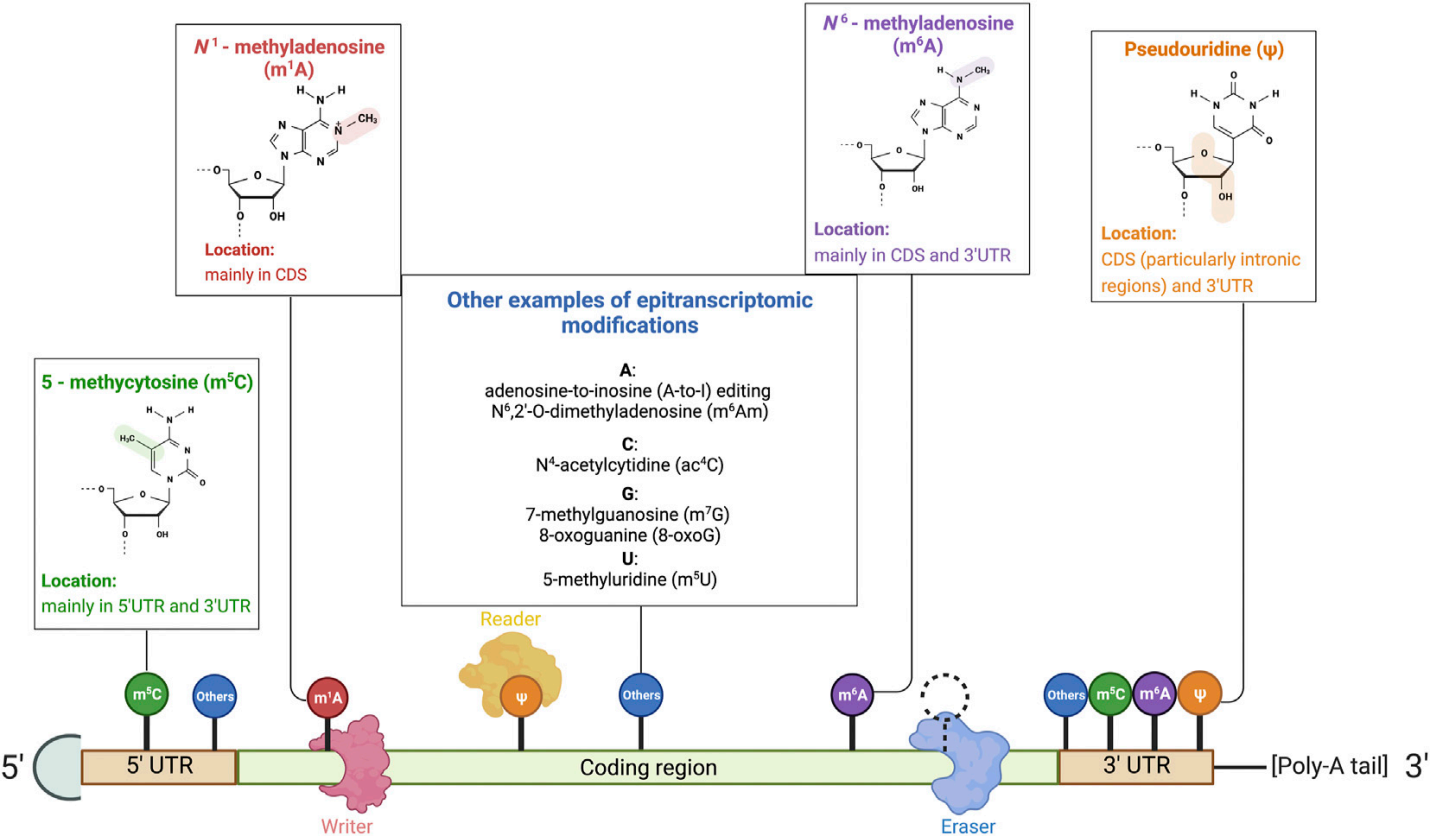
Illustration of RNA-modifying proteins on mRNA and common RNA modifications. Common base modifications include N^6^–methyladenosine (m^6^A), N^1^–methyladenosine (m^1^A), pseudouridine (ψ), and 5–methycytosine (m^5^C), to name but a few. Less common modifications are also listed in the illustration. RNA-modifying proteins that govern the expression of the mRNA transcript by manipulating epitranscriptomic sites include (1) writers that deposit RNA modifications, (2) erasers that remove the epitranscriptomics modifications, and (3) readers that are recruited and recognize the modifications to alter the fate of transcripts. Reprinted from “Common eukaryotic mRNA modifications”, by BioRender.com (2020). Retrieved from https://app.biorender.com/biorender-templates.

RNAWRE, which was constructed in 2020 by Nie et al. (2020) and apropos to mention, comprises more than 2000 manually curated writers, erasers, and readers. RMP regulation determines whether the previously mentioned epitranscriptomic signatures are installed, removed, or recognized. By dint of Table 1 summary and Figure 1 illustration, types of epitranscriptomic marks and their respective RMPs will not be outlined thoroughly in paragraphs. The concept of how these epitranscriptomic marks and RMP expression affect the existence and severity of ferroptosis will be discussed in later parts and illustrated in the compiled figures.

**TABLE 1:** Examples of RNA-modifying proteins and associated epitranscriptomic modifications.

Nucleoside execution-on	Type of epitranscriptomic modification	Location (s)	Writer	Reader	Eraser
Adenosine (A)	N6-Methyladenosine (m^6^A)	mRNA, rRNA, snRNA, and tRNA	METTL family members: METTL3-METTL14 heterodimer (assisted by WTAP interacting with VIRMA), METTL4, METTL5–TRMT112 complex, and METTL16	YTHs (YTHDF1/2/3, YTHDC1 with SRSF3, and NXF1 and YTHDC2)	FTO (guided by SFPQ)
			ZC3H13 corporation: ZC3H13-RBM15/RBM15B ZC3H13-WTAP	HNRNP (HNRNPA2B1/C/G)	ALKBH5
			VIRMA/KIAA1429	IGF2BPs (IGF2BP1/2/3)	
			CBLL1/HAKAI	NKAP	
			ZCCHC4		
	N1-Methyladenosine (m1A)	tRNA, mRNA, and rRNA	TRMT family members: TMRT10C and TRMT6-TRMT61A orthologs	YTHDF3	ALKBH1 and ALKBH3
			m1A58 MTase		FTO
	A-to-I editing	mRNA	ADARs (ADAR1/2/3)	—	—
	N6,2′-O-Dimethyladenosine (m^6^Am)	mRNA	PCIF1	—	FTO
Cytidine (C)	5-methylcytosine (m5C)	mRNA, tRNA, rRNA, and ncRNA	NSUNs (NSUN1/2/3/4/5/6/7)	ALYREF	TETs (TET1/2/3)
			DNMT2		
			TRDMT1	YBX1	ALKBH1
			TRM4A/4B		
	N4-Acetylcytosine (ac^4^C)	rRNA and tRNA	NAT10	—	—
	3-Aethylcytidine (m^3^C)	rRNA, tRNA, and mRNA	METTL2/6 (tRNA)	—	ALKBH1
			METTL8 (mRNA)		
Uridine (U)	Pseudouridine (Ψ)	rRNA, tRNA, mRNA, and snRNA	PUS1/2/3/4/6/7/9	—	—
			TRUB1		
			DKC1		
Guanine (G)	7-Methylguanosine (m7G)	mRNA, tRNA, rRNA, and miRNA	METTL1/WDR4	—	—
	N2-methylguanosine (m^2^G)	tRNA and rRNA	rRNA (guanine-N2-)-methyltransferase	—	—
	Queuine (Q)	tRNA	TGT	—	—

### Detecting epitranscriptomics modifications

Even though the adjustments on nucleotides seem slight and minuscule, finding a way to elucidate the epitranscriptomic marks is never simple and uncomplicated. Consecutive efforts are required owing to these nanoscopic modifications down to nucleotides. This review will not focus on the in-depth discussion of epitranscriptomic mark detection, given that such an issue has already been brilliantly reviewed elsewhere (Helm & Motorin, 2017) (Sarkar et al., 2021). Nevertheless, we shall highlight the important ones, including NGS-based techniques or mass spectrometry-based techniques.

#### Next-generation sequencing-based techniques

AlkB-facilitated RNA methylation sequencing (ARM-seq) (Cozen et al., 2015), combines reverse transcription (RT) and enzymatic demethylation and relies on detecting truncations due to existing methylated nucleosides during RT. Localization of truncations from high-throughput sequencing navigates the potential methylated sites in RNA transcripts, except when the reaction reaches RT-silent bases such as pseudouridine, ribothymidine, or m^5^C. Aside from RT-methods, antibody-dependent assays like m^6^A-seq (for m^6^A) or m^1^A-seq (for m^1^A), MeRIP-seq (Dominissini et al., 2015), CLIP-based strategies (Ke et al., 2015), PAR-CLIP–MeRIP (Liu et al., 2015), miCLIP (for methylated nucleosides in RNA) (Hawley & Jaffrey, 2019), and suicide enzyme trap (for identification of methyltransferase targets on RNA strands) (Khoddami & Cairns, 2013) have also revolutionized the epitranscriptomic mark detection. By eliminating the possibility of having RT-arrest and mis-incorporation of nucleosides during RT like RT-based detection, enrichment-based methods stand out with their superb specificity to methylated nucleosides.

#### Mass spectrometry-based techniques

Dating back to 1977, McCloskey and Nishimura were the first to utilize MS to detect tRNA modifications down to nucleoside resolution. The RNA MS regimen relies on enzymatic digestion/reduction of RNA strands to nucleosides/nucleotides with the nucleic acid backbone being eliminated, and the downward workflow is analogous to metabolite MS, including ionizing the compound and deflecting the molecule in an electric field, followed by a magnetic field. The determination of an m/z ratio greatly depends on retention time, molecular mass, and fragmentation patterns in tandem mass spectrometry (MS/MS) for the identification of modification residues (Helm & Motorin, 2017). Variations of MS include combination with liquid chromatography purification on RNA fragments *a posteriori* nuclease such as RNase T1 and MC1, followed by electrospray ionization (ESI) and MS/MS, entitled LC-ESI-MS/MS (Yuan, 2017). Two years ago, Wein et al. (2020) constructed an open-source database for documenting RNA MS data named NucleicAcidSearchEngine (NASE). Heiss et al. (2021) have also recreated LC-MS/MS by combining nucleic acid isotope labeling (NAIL) and MS, entitled NAIL-MS, to address the dynamic nature of epitranscriptomic modifications that the currently available MS protocols lack the ability to tackle. Nonetheless, despite the comprehensiveness offered by MS, respective localization of modifications in the RNA environment will be completely lost and irretrievable.

## Ironing out the iron: investigating ferroptosis

The first observation on erastin-induced lethality in engineered Ras-mutant human foreskin fibroblasts discovered distinctive morphological features and biochemical machineries compared to traditional programmed cell death (PCD). *Ferroptosis*, coined in 2012 under the work of Dixon et al. (2012), has shed light on the field of PCD and has, henceforth, attracted heed from cell biologists. Devoid of apoptotic morphological features, such as apoptotic body formation or nuclear fragmentation, ferroptotic cells are characterized by increased mitochondrial densities and reduction of mitochondrial crista that are not observed in the conventional PCD (Li et al., 2020). The discovery of iron chelation also denoted an unprecedented biochemical pathway in regulating ferroptosis. Even so, much of our knowledge in ferroptosis is still not complete nor is satisfactory enough to intervene this mechanistic pathway in the current clinical settings.

In the history of ferroptosis characterization, the pioneering finding of erastin has led to the comprehensive dissection of ferroptosis in recent years. Large-scale screening experiments in surveying the killing effects of a multitude of compounds exerted on cancer cells *via* mitochondrial voltage-dependent anion channels, conducted by Dolma et al. (2003), have directed the very first discovery of erastin. Few years afterward, erastin treatment was investigated, and the results of lipid-related oxidative stress were noticed by Yagoda et al. (2007). The RAS-selective lethal 3 (RSL3) was brought up in 2008 from another large-scale synthetic lethal screening by Yang & Stockwell (2008) in the presence of RAS (therefore, the nomenclature). Dixon et al. (2012) officially entitled this iron-dependent cell death as “*ferroptosis*”. Successful characterization has then propagated more in-depth discoveries, including ferrostatin-1 (fer-1) inhibition of ferroptosis, mitochondria independency (Gaschler et al., 2018), sorafenib induction of ferroptosis (Lachaier et al., 2014) (Louandre et al., 2013), system X_c_^−^ being inhibited by erastin (Dixon et al., 2014) (grounded in the fact that cystine deprivation leads to glutathione-dependent cell death long before the characterization of ferroptosis (Eagle, 1955) (Hinson et al., 2010)), glutathione peroxidase 4 (GPX4) participation (Yang et al., 2014), and enormous regulatory ferroptotic inducers (other than erastin, e.g., DPIs, FIN56, and FINO2) and inhibitors (e.g., iron chelators, vitamin E, SRS8-24, and CA-1).

### Delineating the mechanisms of ferroptosis

**FIGURE 2:**
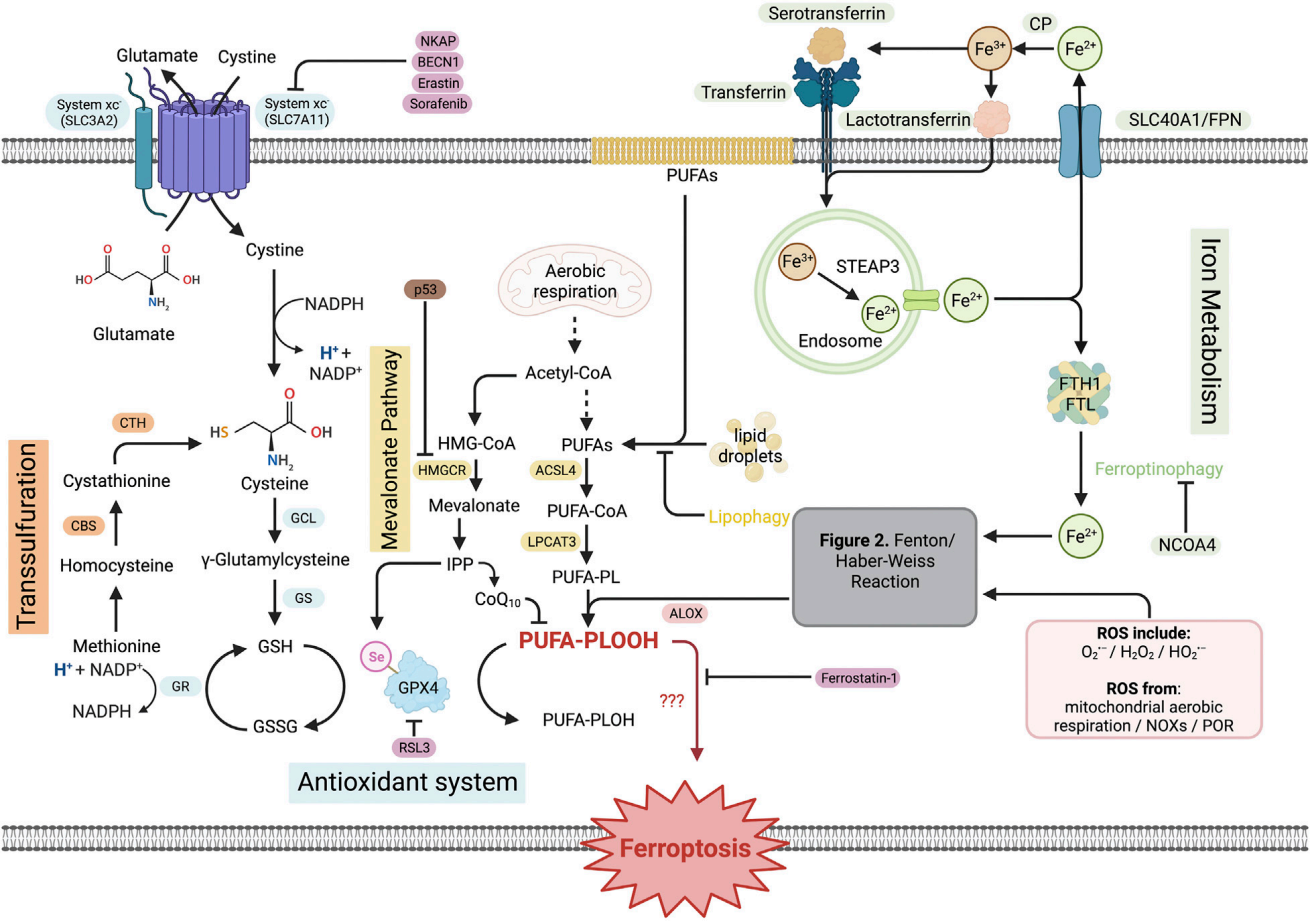
Pathways of ferroptosis. The entirety of ferroptosis signaling is complex and orchestrated by different sub-pathways, along with a multitude of regulatory proteins or substances. The antioxidant system starts with system xc^−^ activity that assists the exchange of cystine and glutamate. Intracellular cystine is converted, in multi-step reactions, to GSH. The transsulfuration reaction starts with conversion of intracellular methionine to cysteine and joins the antioxidant system to enhance GSH production. Lipid ROS production from membrane PUFAs, intracellular lipid droplets, and acetyl-CoA resulted from mitochondrial aerobic respiration, which is negatively regulated by lipophagy, provides predominant lipid source to produce lipid ROS by joining the Fenton/Haber–Weiss reaction. Iron metabolism starts with Fe^3+^ endocytosis initiated by a transferrin receptor, and STEAP3-mediated reduction to Fe^2+^ takes place in endosome. Fe^2+^ joins LIP by FTH1/FTL. Ferritinophagy triggers the release of Fe^2+^ to join intracellular ROS pool and proceeds to the Fenton/Haber–Weiss reaction to produce lipid ROS. Taken together, the PUFA-PLOOH resulting from the reactions induces ferroptotic damage with the mechanism that lacks exactitude. Created with BioRender.com.

Ferroptosis starts with the production of lipid peroxides as a general cellular suicidal program with an iron-mediated oxidative mechanism. Cellular reactions exhibit redox equilibrium, and disruption of redox equilibrium is attributed to the synthesis and accumulation of reactive oxygen species. Definitive ROS, including superoxide anion (O_2_^−^•), hydrogen peroxide (H_2_O_2_), hydroxyl radicals (HO•), hydroperoxides (ROOH), and hydroxyl radicals (ROO•), are formed by partial reduction of oxygen. ROS are generated inevitably from oxidative phosphorylation in mitochondria to cellular respiration, and the endogenous antioxidant system is instrumental to remove the oxidative stress. It has been held as an axiom that ROS accumulation also lays the groundwork of multiple pathologies, given its roles in cellular damage in diabetic cardiomyopathy (Kaludercic & Di Lisa, 2020), atherosclerosis (Yang et al., 2017), neurological complications (Manoharan et al., 2016), and in cell growth, especially in cancers (Aggarwal et al., 2019) (Dias Amoedo et al., 2020) (Tien Kuo & Savaraj, 2006) (Zeng et al., 2021). A detailed mechanistic overview of ferroptosis is illustrated in Figure 2.

**FIGURE 3:**
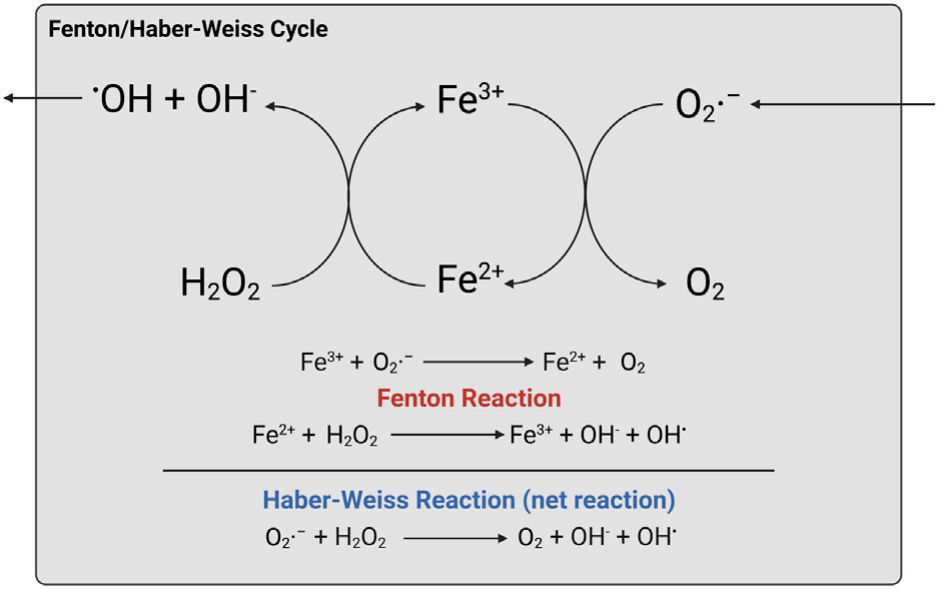
Fenton/Haber–Weiss reaction. Created with BioRender.com.

What lies at the cardiac part of this cellular iron-mediated killing is lipid ROS. The most abundant ROS, superoxide, is generated by cytochrome P450 and NADPH oxidases (NOXs) partial reduction, forming H_2_O_2_ by superoxide dismutase (SOD), and the anions proceed to the production of hydroxyl radicals with the catalytic role of iron. In fact, published articles only documented the iron participation as the Fenton reaction, and the final Haber–Weiss reaction, obtained after balancing chemical equations from Fenton and the others, was less recognized than the Fenton reaction. Ferrous ions (Iron (II) or Fe^2+^) are mainly produced from the labile iron pool (LIP) and upon radical attack to heme groups with iron–sulfur (Fe–S) clusters (Gomez et al., 2014). Oxidation of ferrous to ferric ion (iron (III) or Fe^3+^) facilitates free radical formation from H_2_O_2_, whilst the O_2_^−^• radicals are also oxidized to harmless O_2_ as a net Haber–Weiss reaction. Taken together, the iron-mediated production of hydroxyl radicals is a “superoxide-driven Fenton-catalyzing Haber–Weiss reaction,” or Fenton/Haber–Weiss reaction, as illustrated in Figure 3.

After the Paleoproterozoic Great Oxygenation Event (GOE), lives on the earth were subjected to oxidation readily, especially for polyunsaturated lipids with bis-allylic carbons (Wagner et al., 1994). The victim of such ROS attack in ferroptosis after all the aforementioned series of events is, therefore, polyunsaturated fatty acids (PUFAs). Under normal physiology, PUFAs, including arachidonic acid, eicosapentaenoic acid (EPA), and docosahexaenoic acid (DHA), are situated in the cell membrane. The attack from accumulating free radicals to PUFAs, otherwise named peroxidation reaction, generates phospholipid free radicals (PL•) and, therefore, PUFA-containing-phospholipid hydroperoxides (PL-PUFA (PE)-OOH, PLOOH in short) (Forcina & Dixon, 2019), facilitated by different lipoxygenases (LOXs). It was also demonstrated that depletion of an acyl-CoA synthetase ACSL4 and LPCAT3 esterification enzyme inhibited ferroptosis (Doll et al., 2017) (Yuan et al., 2016). PLOOHs execute the unelucidated last hit to the cell membrane and initiate disruption to cellular integrity, leading to ferroptosis.

The transmembrane cystine/glutamate exchanger commences the work to initiate a ferroptosis-specific antioxidant system. System x_c_^−^, which was found to be inhibited by erastin, serves as an amino acid homeostatic control with the exchange of extracellular L-cystine and intracellular L-glutamate. Dissecting the antiporter, it consists of two subunits, a light chain solute carrier family 7 member 11 (SLC7A11) and a heavy chain subunit SLC family 3 member 2 (SLC3A2), which are targeted by respective inhibitors. Intracellular cysteine from cystine reduction facilitates the production of glutathione (GSH) that is catalyzed by glutamate–cysteine ligase catalytic subunit (GCLC) (which is inhibited by buthionine sulfoximine (BSO)) and then by glutathione synthetase (GSS). The classical redox-associated glutathione system (GSH and oxidized GSH disulfide (GSSG)) comes in to play a role in antioxidant defense, proven back in the 90s (Ceballos-Picot et al., 1996). Glutathione peroxidase 4 (GPX4) protects the cells from ferroptotic death by reducing toxic PLOOHs to PUFA-containing-phospholipid hydroxides (PL-PUFA (PE)-OH, PLOH in short), with the presence of selenium (Liu et al., 2021) and GSH (Ursini & Maiorino, 2020). While PLOHs appear to be non-ferroptogenic (not ferroptosis-inducing), this marks the end of the brief ferroptosis mechanisms as the homeostasis is achieved.

Ferroptosis has been observed in different pathologies. For example, in Alzheimer’s disease that is characterized by prominent brain cell death, β-amyloid plaques and neurofibrillary tangles were investigated, and excess iron accumulation and downregulation of iron exporter, ferroportin1, were observed, thereby explaining the oxidative stress exerted and promoting the AD cognitive impairment (Bao et al., 2021). In renal ischemia/reperfusion injury (IRI), ferroptosis is proven in the mediation of renal tubule-synchronized necrosis, and a novel third-generation ferrostatin 16–86 could rescue or protect the tubular damage that contributes to IRI (Linkermann et al., 2014). In cancer, particularly in colorectal cancer, it was evident that ferroptosis promotes metabolic rewiring, or the Warburg effect, which favors cancer cell growth, as well as suppresses ferroptosis sensitivity by inducing ROS production and activating nuclear factor erythroid 2-related factor 2 (NRF2) (Yuan et al., 2021). These are just few examples that ferroptosis correlates with disease progression, and more details about various pathologies can be found in other good articles such as Jiang et al. (2021b) and Yan et al. (2021) for readers’ reference.

## Ferroptosis and epitranscriptomics: neither two monologues nor a mere duologue

Due to technological advancements in investigating epitranscriptomics and firmer theoretical bedrock on the principle of ferroptosis, both topics are gaining escalating heed from scientists. However, the association between epitranscriptomics and ferroptosis has yet been organized. Thence, with reference to the preliminary background knowledge, we summarize some updates on ferroptosis and epitranscriptomic modifications in recent years and attempt to put a new perspective on the investigation of ferroptosis to facilitate the demystification of any connection between epitranscriptomics and ferroptosis.

### Feed-forward interaction: how do epitranscriptomics shape the niche of ferroptotic homeostasis?

#### Ferroptosis and m^6^A

Being the most characterized epitranscriptomic modification, m^6^A has been widely investigated for its relationship with ferroptosis in different pathological phenomena, including cell cycle, drug resistance, biomarkers, or disease signatures. A couple of m^6^A writers, readers, and erasers have been focused to study as a direct or indirect target to mediate ferroptosis, sorted out in Table 2. METTL14 upregulation resulted from doxorubicin treatment in AC16 cardiomyocytes and neonatal rat ventricle cardiomyocytes, and m^6^A “writing” action was observed to be catalyzed on a sponge lncRNA KCNQ1OT1 for miR-7-5p, which cooperated with RNA-binding protein IGF2BP1 to inhibit miR-7-5p activity, leading to transferrin receptor upregulation and iron uptake increase. Such a phenomenon joins the ferroptotic signaling and increases the opportunity of having lipid peroxidation (Zhuang et al., 2021). Another research echoes with the miR-7-5p and doxorubicin chemoresistance study carried out by Song et al. (2021) on exosomal miR-4443 and cisplatin resistance in non-small cell lung carcinoma. Tantamount to apoptosis, cisplatin simultaneously acts as a dual trigger of apoptosis and ferroptosis to kill cancer cells (Guo et al., 2018). On this groundwork, in tumoral and normal tissue-derived exosomes, their team discovered a distinctive expression level of miR-4443 between cisplatin-sensitive and cisplatin-resistant tissues and cell lines, and further functional and bioinformatics studies confirmed that m^6^A writer METTL3 was negatively regulated by miR-4443 overexpression to lower the m^6^A level on ferroptosis-suppressing protein 1 (FSP1), inhibiting its activity to suppress ferroptosis. Bioinformatics analyses on lncRNAs also revealed m^6^A regulators, namely, FMR1, HNRNPC, METTL16, METTL3, and METTL5, were expressed in higher levels than those in ferroptosis low-risk groups (Jiang, W. et al., 2021a). The aforementioned studies provided evidence that epitranscriptomics are phenomenally involved in ferroptotic disease models, particularly in drug-resistant cancers that have the characteristic to overcome cell death events. As ferroptosis is a new type of PCD, the participation of miRNA, lncRNA, or other types of RNA with distinguished epitranscriptomic features is worth investigating to obtain a complete picture of its disease progress contribution, in order to potentiate clinical relevance for disease manipulation in the future. The theoretical basis on how epitranscriptomics shaped the ferroptosis signaling was also exemplified in pan-cancer *in vitro*, including in hepatocellular carcinoma (Fan et al., 2021), hepatic stellate cells (Shen et al., 2022) (Shen et al., 2021), lung adenocarcinoma (Xu et al., 2022), and glioblastoma (Sun et al., 2022).

**TABLE 2:** Discovered epitranscriptomic marks on ferroptosis-related proteins.

Disease model	Mechanisms in ferroptosis	Epitranscriptomic mark-associated protein	Discovery	Reference
Lung cells (A549)	Lipoxygenase pathway, arachidonic acid metabolic process, and response to selenium ion	m6A reader–YTHDF2	BPQDs increase the global m6A level and decrease ALKBH5 to promote ferroptosis-related pathways	Ruan et al. (2021)
Acute myeloid leukemia cell line (TF-1)	GPX4 antioxidant	m6A eraser–FTO	In-house GNRa-CSP12 sensitized AML cells to TKIs by FTO-m^6^A hypomethylation on GPX4 to promote ferroptosis	Du et al. (2021)
AC16 cardiomyocytes and neonatal rat ventricle cardiomyocytes	Iron uptake ROS production	m^6^A writer–METTL14	Doxorubicin induced METTL14 and lncRNA KCNQ1OT1 to inhibit miR-7-5p, triggering the TFRC increase to promote ferroptosis	Zhuang et al. (2021)
Human hepatic malignant and normal cell lines	Cysteine import	m^6^A writer–METTL14; m^6^A reader–YTHDF2	METTL14 suppression in SLC7A11 and thereafter degradation relied on the YTHDF2‐dependent pathway were observed under hypoxia	Fan et al. (2021)
Malignant and normal lung cell lines	Cysteine import	m6A writer–METTL3; m6A reader–YTHDF1	METTL3 modifies the m^6^A level in SLC7A11 by recruiting YTHDF1 to promote ferroptosis in LUAD.	Xu et al. (2022)
Human liver tissues	Cysteine import	m^6^A writer–METTL4; m6A reader–YTHDF1; m6A eraser–FTO	METTL4 upregulation and FTO downregulation increase global m^6^A level in BECN1 mRNA that originally inhibit SLC7A11, and the YTHDF1 increase promotes BECN1 stability to inhibit cysteine intake and promote ferroptosis in HSCs	Shen et al. (2021)
Mice HSCs	Cysteine import	m6A reader–YTHDF1; m6A eraser–FTO	DHA downregulated FTO to increase m^6^A in BECN1 mRNA, leading to YTHDF1-dependent enhanced stability to inhibit SLC7A11 cysteine–glutamate exchange, promoting HSC ferroptosis	Shen et al. (2022)
Human glioblastoma cell lines (U87MG and U251)	Cysteine import	m^6^A reader–NKAP	NKAP binds to m^6^A in SLC7A11 transcripts and promotes transcriptional splicing and maturation to suppress ferroptosis in glioblastoma cells	Sun et al. (2022)
CRC and adenoma tissues	Ferritinophagy	m6A eraser–ALKBH5	CircRNA cIARS interacts with ALKBH5 to positively regulate ferritinophagy in SF-treated HCC cells	Liu et al. (2020)
BMSCs in mice	Erastin-induced ferroptotic cysteine transport	m5C writer–NSUN5	NSUN5 downregulation is correlated with reduced m^5^C in FTH1/FTL, contributing to ferroptosis	Liu et al. (2022)
Human glioma cell line (U251)	Glutamine metabolism in the antioxidant system	A-to-I editing writer–ADAR	ATXN8OS was found to interact with ADAR and downstream interaction with ferroptosis-related targets is suspected to mediate ferroptosis. These targets include GLS2	Luo et al. (2022)

#### Ferroptosis and other epitranscriptomic marks

A majority of the published articles were m^6^A-based, and there is a huge lack of epitranscriptomic discoveries regarding other marks on ferroptosis. m^5^C is second to m^6^A in terms of the level being explored, and the investigation is still ongoing since we are only scratching the surface of the epitranscriptomic modifications aside from m^6^A (Liu et al., 2022). In fact, one closely related work that is also one of the most recent discoveries bridging epitranscriptomics and ferroptosis was on m^5^C and its exclusive writer NOP2/Sun RNA methyltransferase 5 (NSUN5). In bone marrow-derived mesenchymal stem cells (BMSCs), Liu’s group reported a notable downregulation of NSUN5 in ferroptotic cells and unveiled the enhancement of Fe^2+^ ions in NSUN5 depletion *in vitro*. More importantly, NSUN5 overexpression, which was later confirmed as its methylating action on 5′UTR/3′UTR of ferritin heavy chain/light chain (FTH1/FTL), was correlated with TRAP1 recruitment on FTH1/FTL, a protein that governs the intracellular entry of iron ions, confirmed by LC-MS and co-immunoprecipitation (co-IP). Liu’s group has impacted both the fields of ferroptosis and epitranscriptomics by expanding the discussion to other base modifications other than the predominant m^6^A. Meanwhile, further studies on other disease or cell models, or more superior 3D culture and organoid models, necessitate to be carried out for proof-of-concept.

In addition to m^5^C, in triple-negative breast cancer patients, investigating the tumor microenvironment (TME) guided the discovery of a rare epitranscriptomic feature that serves as a potential biomarker in microniches. Using spatial epitranscriptomic analyses on tumor microniches, Lee et al. (2022) sought to profile A-to-I editome and identified high A-to-I editing in GPX4 variants in IF-stained tissues full-length transcriptome. This result fitted their hypothesis that cancer stem cells (CSCs) contain high A-to-I editing characteristic for their niche shaping, and the future validation work can potentiate the druggability of such epitranscriptomic feature in this ferroptotic-signaling protein.

### Feedback interaction one: how will lipid ROS accumulation potentially influence the nuanced epitranscriptomic features back?

Cellular signaling in biological systems evolved with harmonized crosstalk and attempting to inspect the entirety via a single chronological representation remains laborious to reach the finality. It becomes interesting whether the accumulating lipid ROS being non-eliminated construct a feedback influence on the epitranscriptomic marks. Oxygen atoms in –OH groups and phosphodiester backbone are the most vulnerable to be subjected to chemical damage or oxidation (Liu et al., 2022), and ROS onslaught has demonstrated evidently in mutations (Niedernhofer et al., 2003), cell arrest (Dixon & Stockwell, 2014), and epitranscriptomic induction (Kumar & Mohapatra, 2021). Particularly in cancer, m^6^A induction has been studied and reviewed in response to the production of ROS, and a biphasic and conflicting effect on tumor growth, intriguingly, has been noticed (Chio & Tuveson, 2017) (Yang & Chen, 2021). The potential ROS effect in ferroptosis via epitranscriptomic mediation is hence plausible.

Since the concept of “global m^6^A level can be ROS-induced” was revealed, one ROS-induced post-translational regulation on m^6^A demethylase was discovered recently (Yu et al., 2021). In this study by Yu et al., human cell lines with high m^6^A induced by ROS and determined by m^6^A-seq, were employed to survey the intrinsic mechanism that contributed to the elevation, where SUMOylation in m^6^A demethylase ALKBH5 was found to be associated using comet analysis, a single-cell gel electrophoresis assay that helps determine DNA damage and repair equilibrium at a single cell level. Particularly, SUMOylation-deficiency in ALKBH5 led to weakened DNA repair in H_2_O_2_-induced DNA damage, in other words, SUMOylation in ALKBH5 is essential in the increase of global m^6^A level by limiting the activity of m^6^A erasers. As ROS also joins the ferroptotic signaling and can lead to ferroptotic cell death, how ROS can potentially construct a stressful environment and add on epitranscriptomic modifications of ferroptosis proteins remains to be extrapolated. Having a feedback loop discovered that thrusts in the cell death process offer a great potential to manipulate the pathways, and the prospects of targeting ferroptosis in therapeutic settings await.

### Feedback interaction two: how does iron imbalance contribute to an epitranscriptomic mark level?

Dixon et al. (2012) extensively acknowledged the importance of iron in its mediation to the PCD event by coining the “ferro-” in the nomenclature of the iron-driven cell death, ferroptosis, assisted by the Nomenclature Committee of Cell Death (NCCD). Before then, prominent iron overload was observed among pathologies, such as hereditary hemochromatosis, along with the complications manifested, including organ damage, hypothyroidism, and hypogonadism. Managing iron homeostasis, thence, is necessitated from a medical standpoint, combined with the fact that ferroptosis is also dependent on intracellular iron status. In addition, *en route* to the research on how important iron to ferroptosis is, we also discovered some connections between iron and epitranscriptomic marks upon rummaging articles. We aimed to address the potential association of iron status and epitranscriptomics in ferroptosis and provided upcoming possible research directions to facilitate the elucidation of this mystery.

The fact that heme groups and Fe–S clusters are frequently under the attack of various kinds of ROS is well known (Imlay, 2006). This increases the intracellular level of Fe^2+^ apart from the LIP, though the LIP serves as the predominant source of Fe^2+^. In fact, perturbations of epitranscriptomics that affect the iron level or iron metabolism have been shown via some direct studies. In a hypopharyngeal squamous cell carcinoma (HPSCC) study by Ye et al. (2020), transcriptomic analyses including m^6^A-seq, RNA-seq, and RIP-seq identified m^6^A reader YTHDF1’s downstream target transferrin receptor (TFRC), simultaneously linking to poor prognosis in postoperative platinum-based chemoradiotherapy (CCT) or radiation patients in an m^6^A-dependent manner. HPSCC patients with intratumorally elevated Fe^2+^ were also shown upregulated YTHDF1 expression, and knockdown YTHDF1 in HPSCC cells proved the suppression of cell proliferation and migration ability. Taken together, as YTHDF1 modifies TFRC mRNA in cytosol and modulates transcriptomic stability and fate, relationships between an RMP and an iron metabolism participant were speculated by this pioneering work. Additionally, a pancreatic ductal adenocarcinoma (PDAC) study by Huang et al. (2021) aimed at elucidating the connection of ALKBH5 and iron metabolism, concretely on mRNAs encoding ubiquitin ligase FBXL5 and iron importers SLC25A28 and SLC25A37. ALKBH5 was identified to be mechanistically associated to the RNA decay event for FBXL5, and the team has divulged its unique prognostic ability among multiple m^6^A regulators analyzed in the study. Considering FBXL5-IRP2 serves as the cardinal part to iron metabolism (Wang et al., 2020), this study adds on the evidence of connecting epitranscriptomic-mediated iron metabolism since the bridge between FBXL5 and ALKBH5 can now be surmised through transcriptomic analyses, and further validation work awaits to confirm.

#### Prospect and unaddressed questions

Yet, tracing back to the fact that excess intracellular iron leads to disturbed redox imbalance, and hence impaired cellular metabolism, we shall also pay heed to the crosstalk between iron metabolism and epitranscriptomics. Despite limited direct studies on the biological functions, some RMPs are reported to be affected by iron levels. m^6^A demethylase ALKBH5 was Fe^2+^-dependent, proven in an optimization research study for downstream screening work by Li et al. (2016). Therefore, it leaves us with the following questions on 1) how much iron level deviation intracellularly can drive impaired ALKBH5 function; 2) how much Fe^2+^ perturbations can lead to redox imbalance, followed by the epitranscriptomic mark writing on RMPS that constitutes to a crosstalk signaling; and 3) what are the signaling paradigms required for iron-driven/ROS-induced epitranscriptomic mark writing and the potential involvement to ferroptosis. There are still many unsolved questions that build around the biological or biomedical conjectures on ferroptosis and epitranscriptomics that begin with iron imbalance and ROS induction. Addressing these outstanding questions shall help determine the direct involvement of distinct components in ferroptosis.

## Concluding remarks

In retrospect, investigating a new topic in science has always been regarded as preposterous at the beginning, and the journey of vindication seems to be life-long and with collaborative efforts. Epitranscriptomics have indeed experienced a dejected period due to the lack of technological advancement, but the value *per se* is tantamount to epigenetic modifications owing to its importance in governing the ultimate phenotype of a gene. It is hard for us to ignore the participation of such element being pervasive on gene expression in ferroptosis, a new type of PCD discovered just in recent decades, and is still being explored for its potential clinical relevance. As the evidence regarding epitranscriptomics and ferroptosis began to pile up, with the associated indirect studies on the passengers of both, RMPs or lipid ROS, *videlicet*, we offered additional perspectives for readers to define the pathways of ferroptosis with respect to epitranscriptomic modifications, and thus to provide foreseeable opportunities toward comprehensiveness of such topics.
